# Role of lymphadenectomy in advanced-stage ovarian cancer: a meta-analysis

**DOI:** 10.3389/fsurg.2024.1481625

**Published:** 2024-11-27

**Authors:** Carlo Ronsini, Francesca Pasanisi, Giada Andreoli, Pasquale De Franciscis, Luigi Cobellis, Giuseppe Vizzielli, Stefano Restaino, Paola Romeo, Vittorio Palmara, Stefano Cianci

**Affiliations:** ^1^ Department of Woman, Child and General and Specialized Surgery, University of Campania “Luigi Vanvitelli”, Naples, Italy; ^2^ Clinic of Obstetrics and Gynecology, 'Santa Maria della Misericordia' University Hospital, Azienda Sanitaria Universitaria Friuli Centrale, Udine, Italy; ^3^ Department of Medicine, University of Udine, Udine, Italy; ^4^ Unit of Gynecology and Obstetrics, Department of Human Pathology of Adult and Childhood “G. Barresi”, University of Messina, Messina, Italy

**Keywords:** lymphadenectomy, ovarian cancer, advanced stage, overall survival, disease free survival

## Abstract

**Background:**

Epithelial Ovarian Cancer is one of the most lethal cancers among gynecologic malignancies. The disease metastasizes mainly through the peritoneal spread in the abdomen and through the lymphatic system. Lymph node involvement is present in 48% up to 75% of cases of advanced-stage ovarian cancer (ASOC). In this context, the aim of our study is to analyze the current literature on the topic and to investigate survival outcomes in patients affected by advanced-stage ovarian cancer undergoing lymphadenectomy.

**Methods:**

Following the recommendations in the Preferred Reporting Items for Systematic Reviews and Meta-Analyses (PRISMA) statement, we systematically searched the Pubmed and Scopus databases in June 2022 since the first publication. We made no limitations on the country. We included the studies containing disease-free survival (DFS) and Overall Survival (OS) data. Only comparative studies with a direct comparison between Lymphadenectomy and its avoidance were included for meta-analysis.

**Results:**

18 studies fulfilled the inclusion criteria. The overall OS, DFS, and RR were comparable in the studies. 26965 patients were enrolled in the meta-analysis. Patients were analyzed concerning OS and DFS. Meta-analysis highlighted statistically significant higher OS than the lymphadenectomy group (RR 1.31 [95% CI 1.16–1.48] *p* < .00001), and no statistically different DFS RR 1.23 [95% CI 0.82–1.92] *p* = 0.25).

**Conclusion:**

Our analysis showed a protective role of lymphadenectomy in advanced ovarian cancer, with a reduction in death risk.

**Systematic Review Registration:** www.crd.york.ac.uk/prospero/display_record.php?ID=CRD42022341646, Identifier CRD42022341646.

## 1 Introduction

Epithelial Ovarian Cancer (EOC), although in a downward trend in the last years, remains one of the most lethal cancers among gynecologic malignancies (1). The main reason is related to the high incidence of diagnosis at advanced stages, usually associated to peritoneal spread and organ metastasis. This is mainly related to the absence of specific symptoms at early stages and most of diagnoses at this time are incidental following routine examinations (2).

The gold standard for ASOC treatment is primary surgery to completely remove all visible diseases, followed by platinum and taxanes-based adjuvant chemotherapy (3).

However, one of the main debated arguments related to ASOC treatment is the way of spreading, leading to lymphatic metastasis. The literature reports the lymph node involvement ranges between 48% and 75%, even based on the histologic sub-type of primary disease (4).

The available guidelines for ASOC surgical treatment suggest total hysterectomy with bilateral salpingo- oophorectomy, plus complete resection of peritoneal deposits and the resection of macroscopically diseased nodes. The surgery aims to reach the absence of residual tumor (5).

One of the main reasons for controversy is represented by the necessity of systematic lymphadenectomy in order to remove the metastatic nodes completely. Worth considering the difficulties in macroscopically distinguishing the extent of disease, especially in the case of patients submitted to neo-adjuvant chemotherapy. Moreover, the procedure may be associated to several post operative complications, impacting the quality of life of the patient. The argument is well represented in literature with randomized controlled trials (RCTs) and retrospective studies, but no unique and definitive conclusions are available. On this base, the real benefits of extensive lymph nodes removal need to be better investigated (6–8). The role of systematic lymphadenectomy remains controversial, both for advanced and early stages, since the results from different studies reported discordant conclusions concerning disease-free survival (DFS) and overall survival (OS) outcomes. The heterogeneity of the available studies represents the other bias, since most of the works do not stratify patients on the stage or histotype (9–11).

The current meta-analysis aims to investigate and compare survival outcomes related to systematic lymphadenectomy or its avoidance in a specific subgroup of patients represented by women affected by EOC at advanced resectable stages.

## 2 Material and methods

The methods for this study were specified *a priori* based on the recommendations in the Preferred Reporting Items for Systematic Reviews and Meta-Analyses (PRISMA) statement (12). We registered the Review to the PROSPERO site for meta-analysis with protocol number CRD42022341646.

### 2.1 Search method

We performed a systematic search for articles about Lymphadenectomy during debulking surgery of Stages III and IV (FIGO 2014) of Epithelial Ovarian Cancer in PubMed Database, and Scopus Database in July 2024 since the first publication. We made no restrictions on the country. We considered only English entirely published studies.

### 2.2 Study selection

**Figure 1:**
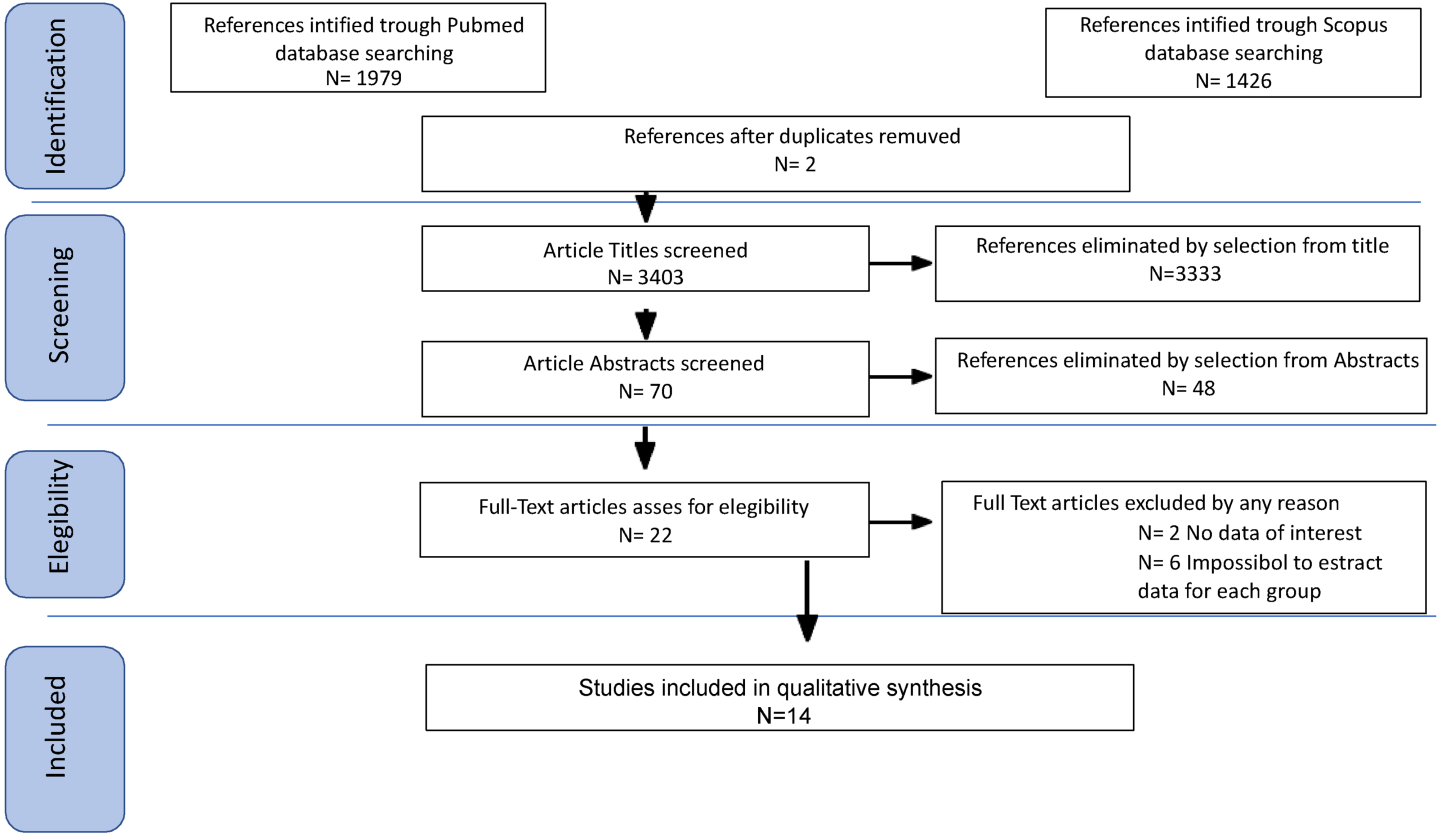
Preferred reporting items for systematic reviews and meta-analyses (PRISMA) flowchart.

Study selection was made independently by FP and PDF. In case of discrepancy, CR decided on inclusion or exclusion. Inclusion criteria were: (1) studies that included patients with epithelial ovarian cancer (EOC) stage FIGO IIB or greater undergoing Primary or Interval Debulking Surgery (PDS or IDS); (2) studies comparing outcomes of interest in patients undergoing systematic pelvic and paraaortic lymphadenectomy and patients who did not - in the latter group patients whose lymph node status has not been assessed were included, or women who received only nodal biopsy or removal of bulky nodes [only in Benedetti Panici 2005 (6)]; (3) studies that reported at least one outcome of interest (Overall Survival (OS); Disease-Free Survival (DFS); Recurrence rate (RR)); (4) Studies that had equal distribution in residual tumor in both groups of their population, (5) peer-reviewed articles published originally. We excluded non-original studies, preclinical trials, animal trials, abstract-only publications, and articles in a language other than English. If possible, the authors of studies that were only published as congress abstracts were tried to be contacted via email and asked to provide their data. We mentioned the studies selected and all reasons for exclusion in the Preferred Reporting Items for Systematic Reviews and Meta-Analyses (PRISMA) flowchart (Figure 1). We assessed all included studies regarding potential conflicts of interest.

### 2.3 Statistical analysis

Heterogeneity among the studies was tested using the Chi-square test and I-square tests (13). The risk rate (RR) and 95% confidence intervals (CI) were used for dichotomous variables. Fixed-effect models conducted statistical analysis without significant heterogeneity (*I*^2^ < 50%), or random-effect models if *I*^2^ > 50%. DFS, RR, and OS were used as clinical outcomes. A sensitivity analysis was performed by means of metainference to estimate the weight of each study included in the heterogeneity. An Egger's regression was conducted to assess the publication bias and skewness of the studies. In each study, Disease-free survival was defined as the time elapsed between surgery and recurrence or the date of the last follow-up. Overall survival has been defined as the time elapsed between surgery and death for disease or the last follow-up. Recurrence Rate has been defined as the ratio of patients relapsing over the total of patients enrolled, during the follow-up period. Chi-square tests were used to compare continuous variables. Review Manager version 5.4.1 (REVman 5.4.1), R software (RStudio version 2024.04.02) and IBM Statistical Package for Social Science (IBM SPSS vers 25.0) for MAC were used for statistic calculation. For all performed analyses, a *p*-value <0.05 was considered significant.

### 2.4 Quality assessment

We assessed the quality of the included studies using the Newcastle–Ottawa scale (NOS) (14). This assessment scale uses three broad factors (selection, comparability, and exposure), with the scores ranging from 0 (lowest quality) to 8 (best quality). Two authors (MCS and II) independently rated the study's quality. Any disagreement was subsequently resolved by discussion or consultation with CR. We reported NOS Scale in Appendix A. We used a funnel plot analysis to assess publication bias. We used Egger's regression test to determine the asymmetry of funnel plots.

## 3 Results

### 3.1 Characteristics

After the database search, 3,405 articles matched the searching criteria. After removing records with no full text, duplicates, and wrong study designs (e.g., reviews), 23 studies were suitable for eligibility. 19 of them were comparative studies between systematic pelvic and paraaortic lymphadenectomy and its avoidance, including in the second group patients whose lymph node status has not been assessed, or women who received only nodal biopsy or removal of bulky nodes. Comparative works were included in quantitative analysis (6, 8, 15–31) (Figure 1). Table 1 summarizes the main characteristics of the selected articles, such as the publication year, the study design, the population's FIGO Stage, the number of participants, and the mean number of lymph nodes retrieved. The quality of all studies was assessed by NOS (14) (Appendix A). Overall, the publication years ranged from 1995 to 2024, with the last study published in July 2024 (25). In total, 18,059 patients from FIGO stage IIB to IV with resectable disease were enrolled; among those 11,947 underwent lymphadenectomy and 6,112 did not. The follow-up period ranged from 22 to 68.4 months on average. The mean number of lymph nodes retrieved in the lymphadenectomy group ranged from 4 to 57.

**Table 1:** Studies characteristics.

Name	Country	Study design	Study year	FIGO stage/Population	N of participant (LND vs. NO-LND)	LNs removed n. (range)	Mean FUP months
Abe et al. (15)	Japan	Retrospective multicenter cohort study	2001–2005	III–IV	56 (28 vs. 28)	33 (9–80)	31
Aletti et al. (16)	USA	Retrospective monocenter cohort study	1994–1998	IIIC–IV	219 (61 vs. 158)	21 (3–48)	36
Panici et al. (6)	Italy	Retrospective monocenter cohort study	1991–2003	IIIB–IV	427 (216 vs. 211)	51.5 (41–70)	68.4
Bund et al. (17)	France	Retrospective multicenter cohort study	2000–2017	III–IV	255 (155 vs. 100)	28 (N/A)	N/A
Chan et al. (18)	USA	Retrospective multicenter cohort study	1988–2001	III–IV	13,918 (4260 vs. 9,658)	6 (1–54)	22
Chang et al. (19)	Korea	Retrospective monocenter cohort study	2000–2011	IIIC	189 (135 vs. 54)	18 (3–57)	N/A
Eoh et al. (20)	Korea	Retrospective monocenter cohort study	2009–2015	IIIC–IV	133 (65 vs. 68)	4 (1–9)	N/A
Fang et al. (21)	China	Retrospective monocenter cohort study	2004–2013	III–IV	410 (210 vs. 200)	N/A	68.4
Fukasawa et al. (22)	Japan	Retrospective monocenter cohort study	1986–1991	IIIB–C	69 (33 vs. 36)	N/A	N/A
Gao et al. (23)	China	Retrospective monocenter cohort study	2010–2020	IIB–IVB	80 (57 vs. 23)	N/A	60
Harter et al. (8)	Germany	Prospective multicenter randomized study	2008–2012	IIB–IV	647 (323 vs. 324)	57 (N/A)	60
Ikeda et al. (24)	Japan	Retrospective multicenter cohort study	1986–2017	IIB–IV	335 (170 vs. 165)	N/A	49.8
Nasidius et al. (25)	USA	Retrospective monocenter cohort study	2005–2010	III–IV	1,060 (125 vs. 935)	29 (20–72)	38.2
Paik et al. (26)	Korea	Retrospective monocenter cohort study	2002–2013	III–IV	261 (135 vs. 126)	17 (8–51)	48
Sakai et al. (27)	Japan	Prospective monocenter case-control study	1986–2009	III–IV	180 (87 vs. 93)	N/A	49.4
Scarabelli et al. (28)	Italy	Prospective non-randomized monocenter cohort study	1985–1993	IIIC–IV	142 (98 vs. 44)	47 (35–79)	33
Schwartz et al. (29)	France	Retrospective multicenter cohort study	1998–2012	III–IV	101 (54 vs. 47)	13.5 (8–23)	34
Song and Gao (30)	China	Retrospective monocenter cohort study	1996–2016	IIIC–IV	330 (263 vs. 67)	19.5 (6–36)	65
Yin and Wang (31)	China	Retrospective multicenter database analysis	2010–2019	III–IV	10,184 (5,472 vs. 4,712)	N/A	N/A

LNs, lymph nodes; LND, lymphadenectomy; FIGO, International Federation of Gynecology and Obstetrics; NA, not applicable; FUP, follow up.

### 3.2 Outcomes

All the 18,059 patients were included in the meta-analysis. 13 selected studies presented 5 years of DFS data. 17 studies presented 5 years OS data. The overall 5Y-DFS for patients who underwent lymphadenectomy ranged from 2% to 65%, Vs a range from 0 to 52% for patients who did not. Also, 5Y-OS for patients who underwent lymphadenectomy ranged from 19% to 76%, Vs a range from 21% to 78% for patients who did not. Those results are summarized in Table 2.

**Table 2:** Oncological outcome DFS and OS.

Name	No LND 5Y DFS* (%)	LND 5Y DFS* (%)	*P*	No LND 5Y OS° (%)	LND 5Y OS° (%)	*p*
Abe et al. (15)	52	30	0.48	66	65	0.71
Aletti et al. (16)	NR	NR	NR	31	50	0.01
Panici et al. (6)	21.6	31.2	0.01	47	48.5	0.85
Bund et al. (17)	7	8	0.48	21	19	0.73
Chan et al. (18)	NR	NR	NR	26.1	45	<0.001
Chang et al. (19)	NR	NR	NR	38	57	<0.01
Eoh et al. (20)	0	7	0.74	27	58	<0.001
Fang et al. (21)	0	2	0.214	78	76	0.385
Fukasawa et al. (22)	20	65	NR	NR	NR	NR
Gao et al. (23)	NR	NR	NR	46.6	57	0,351
Harter et al. (8)	30	25	0,29	56	54	0,65
Ikeda et al. (24)	28.1	39.9	0.006	51.6	61.5	0.007
Nasidius et al. (25)	NR	NR	NR	56.1	56.8	0.4
Paik et al. (26)	20	23	0.505	40	65	0.002
Sakai et al. (27)	46.7	41.9	0.658	62.9	59	0.853
Scarabelli et al. (28)	9	48	0.02	NR	NR	NR
Schwartz et al. (29)	5	4	0.17	50	40	0.088
Song and Gao (30)	20	8	0.049	60	54	0.566
Yin and Wang (31)	NR	NR	NR	37.49	43.41	<0.001

DFS, disease free survival; OS, overall survival; NR, not reported; LND: lymphadenectomy.

In 8 studies, we were also able to evaluate data about RR, which ranged from 44.5% to 78.5% for the Lymphadenectomy group, vs. 49.3% to 83.3% for patients who did not receive lymphadenectomy, as shown in Table 3.

**Table 3:** Oncological outcome recurrence rate.

Name	No LND recurrence rate (%)	LND recurrence rate (%)	*p*
Panici et al. (6)	69.2	62.5	NR
Bund et al. (17)	58	44.5	0.2
Chang et al. (19)	83.3	48.1	NR
Eoh et al. (20)	80.9	78.5	0.729
Fukasawa et al. (22)	55	45	NR
Ikeda et al. (24)	71	60	NR
Sakai et al. (27)	49.5	60.9	NR
Song and Gao (30)	70.1	68	NR

LND, lymphadenectomy.

### 3.3 Meta-analysis

The 19 studies comparing systematic Lymphadenectomy and its avoidance were enrolled in the meta-analysis. A total of 28,826 patients were analyzed for the OS. 11,786 patients in the Lymphadenectomy arm were compared with 17,040 patients who did not undergo lymphadenectomy. Because of the high heterogeneity (*I*^2^ > 50%; *p* < .00001), a random-effects model was applied.

**Figure 2:**
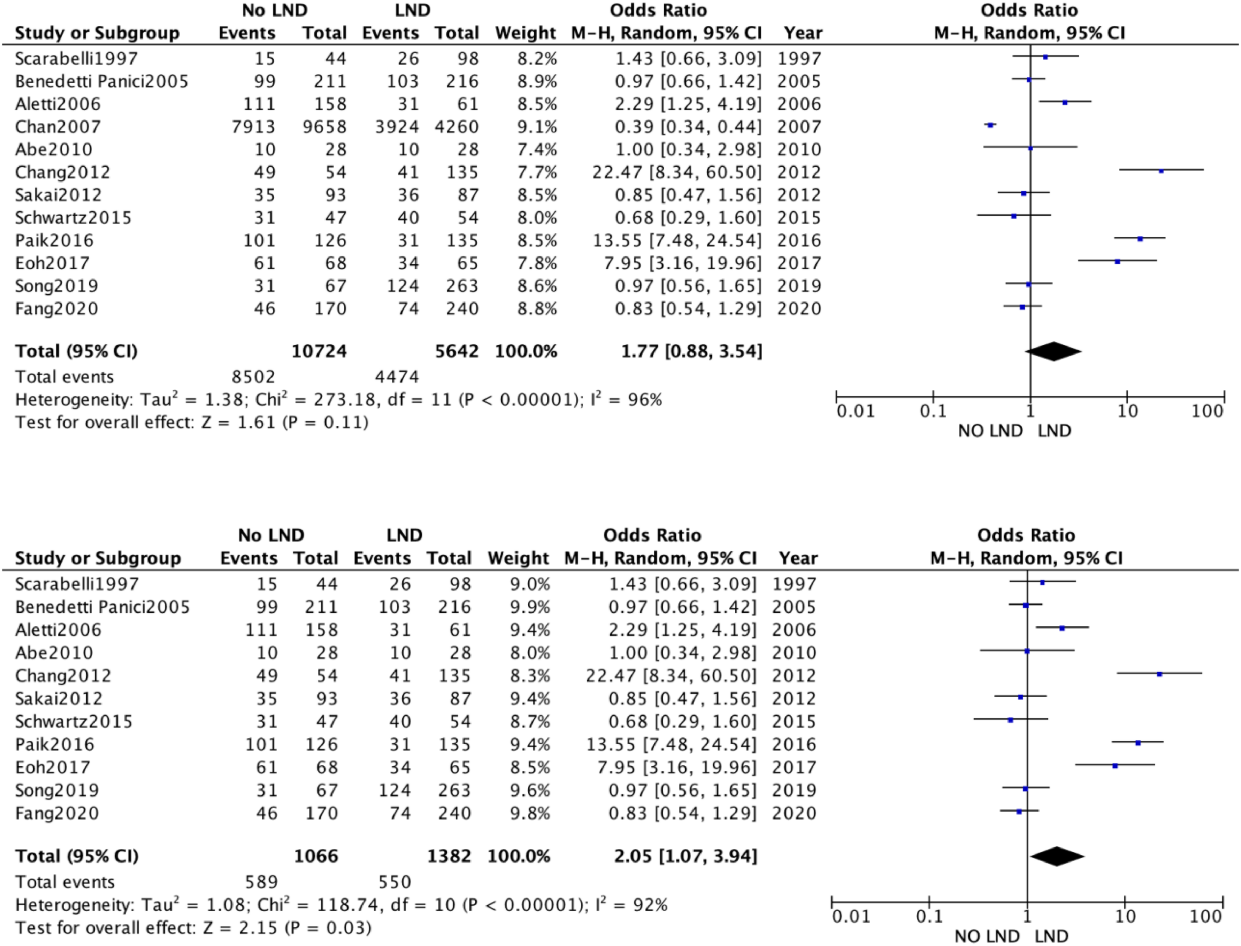
**(A)** Overall survival. **(B)** Overall Survival Subgroup.

The lymphadenectomy group showed a statistically significant higher OS than the No-lymphadenectomy group [RR 1.28 (95% CI 1.14–1.44) *p* < .00001] (Figure 2).

We conducted a sensitivity analysis with estimation of the weight of each included study on heterogeneity. The best result was obtained by excluding the by Chang et al. The results were statistically significant and in line with previous [RR 1.28 (95% CI 1.08–1.52) *p* < .00001] (Figure 2A).

A second analysis concerning DFS outcome was performed. A total of 3,346 women were analyzed. 1,837 patients in the Lymphadenectomy arm were compared with 1,509 patients who did not undergo lymphadenectomy. Because of the high heterogeneity (*I*^2^ > 50%; *p* < .00001), a random-effects model was applied.

**Figure 3:**
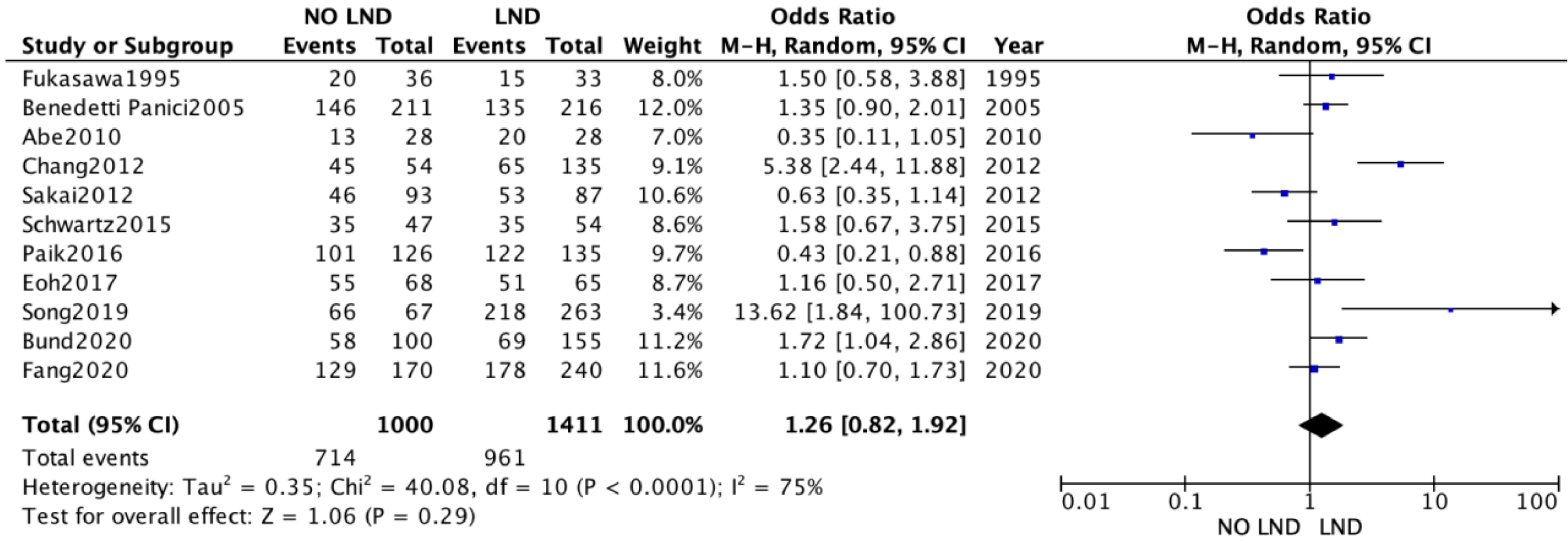
Disease free survival.

DFS in the two groups was non-statistically significantly equal, with an RR's CI which embraced the neutral value [RR 1.23 (95% CI 0.82–1.92) *p* = 0.25] (Figure 3).

## 4 Discussion

Lymphadenectomy at the time of debulking surgery for ovarian cancer treatment remains a debated argument. It is still not clear the benefits in terms of DFS and OS both for the early stage and for the advanced stage. The importance acquires relevance, considering the complications related to this surgical procedure as vascular injury, lymphocele, increased risk of infection and sepsis. These complications could interfere with the timing of chemotherapy treatment and consequently with oncologic outcomes.

The importance of the early start of systemic treatment was demonstrated by Manher et al., who reported that a delay of 7 days in beginning chemotherapy resulted in an 8.7% increase of mortality in patients with complete surgical resection (32). This means that the procedures performed should always be justified and useful. In this context, the real benefit and indication for lymphadenectomy should be continually addressed.

Other aspects that should be included in this evaluation are factors that enhance the intra- and post-operatory risks, such as the presence of metastatic lymph nodes, the age of patients, vasculopathy, and general comorbidity. The main goal at the time of debulking should indeed be the complete removal of all visible disease, and the literature confirms the importance of the absence of residual disease in terms of oncological outcomes (33, 34). However, it is clear that retroperitoneal bulky disease also needs to be removed. The studies focused on this aspect reported different and sometimes controversial conclusions. The two main available trials reported interesting results that could strengthen our previous affirmations (6, 8).

The randomized trial by Benedetti Panici et Al. (6) was aimed to determine the impact of selected resection of only bulky pelvic and para-aortic nodes vs. systematic lymphadenectomy for ASOC treatment. The results obtained demonstrated that systematic lymphadenectomy gives not any add-on OS but only in DFS. However, the systematic lymphadenectomy group required more transfusions than the other group.

The other available randomized trial by Harter et Al. (8) - the LION trial - compared two groups of ASOC - undergoing Primary Debulking Surgery - with macroscopically normal pelvic and para-aortic lymph nodes submitted or not to lymphadenectomy. The results confirmed that systematic pelvic and paraaortic lymphadenectomy was associated with a higher incidence of post-operatory complications without any advantages in overall and progression-free survival.

The results of the aforementioned randomized clinical trials are in line with the data of the CARACO trial's abstract (35), recently presented at the 2024 ASCO Annual Meeting II, which analysed OS and PFS in ASOC patients undergoing systematic lymphadenectomy or not, in the context of interval surgery. We are awaiting the final results, in order to eventually update our meta-analysis.

These data demonstrated that lymphadenectomy is not necessary for ASOC with macroscopically normal lymph nodes, even if some not randomized studies reported opposite results affirming that lymph node dissection gives advantages in DFS and OS.

The importance of the aforementioned works (6, 8, 35) is related to the fact that most of the existing literature on the topic is made of not randomized trials. Many of these works, including large numbers of patients, have reported higher survival rates for patients undergoing systematic lymphadenectomy (18, 31). However, non-randomized studies are predisposed to several biases.

Basing on these data, the correct management of lymphadenectomy for ASOC remains unclear. The unique concordant data is that systematic lymphadenectomy for macroscopically regular nodes is unnecessary, and the balance of risks/benefits seems to be pending to a higher risk of complications (36). Some authors suggest that the risk of occult metastatic cells not being macroscopically detectable could be overlapped by systematic chemotherapy that does not influence oncological outcomes (37).

The main doubt remains about the approach of enlarged lymph nodes. The main point is to define which patients could benefit more from radical lymphadenectomy. In the case of fit patients without relevant comorbidities, the radicality is justified to obtain no residual intra-abdominal disease. However, the real benefit remains controversial for patients affected by significant morbidities or aged patients. The removal of selected enlarged lymph nodes could be the right compromise even if it is not always applicable cause often, the metastatic lymph nodes, are conglomerate forming a sort of package with the consequent need to remove all regional lymph nodes with enhanced intra and post-operatory risk (38). In this context, the actual indications of radical lymphadenectomy should be better addressed. Our analysis aimed to obtain some answers to these questions to reach some indications to adopt based on different cases.

The quantitative analysis of the data we collected does show a clinical advantage in performing lymphadenectomy in ASOC, showing a statistically significant better OS [RR 1.31 (95% CI 1.16–1.48) *p* < .00001]. This finding, however, is vitiated by the great weight exerted by two retrospective studies (18, 31), whose data were extracted from multiple databases, with no indication of the accuracy or overlap of the data.

The data obtained from our meta-analysis overlapped with two available randomized trials as the OS was not different in the two study groups (Lymphadenectomy vs. no nodal biopsy).

DFS analysis yielded results that were not statistically conclusive and did not favor either approach.

However, the meta-analysis showed a trend favoring lymphadenectomy, which future investigations could confirm. Although, it is important to consider that the data of our analysis should be interpreted in light of the significant limitations due to the predominance of retrospective studies, the inability to perform subgroup analyses, and possible interaction tests.

Our results raise some other questions instead of giving definitive answers as the results are opposite to those of randomized available trials. Our data are not unique in literature cause some interesting data merged if we compare our data with the most recent meta-analysis available. The meta-analysis from Chiyoda et Al. (39) reported some similarity with our study because they confirmed the advantages of lymphadenectomy in terms of OS even if early-stage ovarian cancer was included in the analysis. The other meta-analysis from Purwar et Al. instead reported opposite results without a difference in OS between the two groups with a positive trend without the statistical significance of PFS. However, the study included only three randomized studies excluding other series studies (40).

Based on these numbers the role of lymphadenectomy remains controversial, especially in high-risk patients. Another consideration is the lack of stratification of the results analyzed. Most of the studies do not differentiate results basing on patients performance status, age and comorbidities. It may be taken for granted that in retrospective analysis most of the patients who underwent radical surgery were the ones that have been considered fit for this procedure. Moreover, most of the studies do not specify if the surgery was a PDS or an IDS, nor if patients received neoadjuvant Chemotherapy and which protocol. Then, a stratification on tumor hystotype is absent in most works. Some recent authors analyzed oncological outcomes only in a specific hystotype (23, 24).

Therefore, the available results are inconsistent and do not allow for the provision of definitive conclusions. The unique concordant data between different studies is that lymphadenectomy can increase intra and post-operatory complications that can influence the treatment course. Consequentially it is essential to evaluate every single case based on clinical characteristics, make a risk/benefit balance, and then decide on the surgery tailored to patient characteristics.

The role of lymphadenectomy remains controversial not only for ASOC but even for early stages. As reported in a recent study which even for stages I and II, the lymphadenectomy did not influence the OS (9).

The next future will open new therapeutic strategies both from surgery and for medical treatment. For example, experimental approaches to reduce the invasiveness of lymphadenectomy during ovarian cancer surgery are available. A research group reported promising data on the feasibility of sentinel node biopsy for ovarian cancer staging (41).

Moreover, reducing invasiveness could be achieved even by improving systemic treatment. The efficacy of precision medicine in the future could allow reducing the radicality of surgery required to maintain and even improve oncologic outcomes.

Our study finds its strength in the systematic search of all the work produced in the literature and the many enrolled patients. In any case, the different years in which the studies were conducted, the different inclusion criteria, and the predominance of retrospective studies limit its effectiveness. However, the emerging data prove that there is no unambiguous clarity on the role of lymphadenectomy in ASOC and confirm that future randomized trials will be needed to clarify these gray areas.

## 5 Conclusion

Our work highlighted the potential role of lymphadenectomy in improving OS in ASOC patients. In the literature, the role of lymphadenectomy for ASOC is still unclear because the studies available are controversial and prone to biases. Even if some works showed benefits from systematic lymphadenectomy, more randomized studies with selected cohorts need to be carried out. The radicality of surgery should always be addressed and tailored to patients' characteristics since systematic lymphadenectomy may be related to surgical complications. Besides the oncologic outcomes, the best treatment strategies should always consider the quality of life and patients' expectations. Future innovation could play an important role in improving surgical and oncological outcomes.

## Data Availability

The data analyzed in this study is subject to the following licenses/restrictions: Data avaible for every single publication. Requests to access these datasets should be directed to carlo.ronsini@unicampania.

## Appendix

A. New Castel-Ottawa Scale

**Table:**

Comparative Studies						
Name	Country	Study design	Selection	Comparability	Exposure	Tot
Abe et al. (15)	Japan	Retrospective multicenter cohort study	3	1	3	7
Aletti et al. (16)	USA	Retrospective monocenter cohort study	2	1	2	5
Panici et al. (6)	Italy	Retrospective monocenter cohort study	2	1	3	6
Bund et al. (17)	France	Retrospective multicenter cohort study	3	2	2	7
Chan et al. (18)	USA	Retrospective multicenter cohort study	3	1	2	6
Chang et al. (19)	Korea	Retrospective monocenter cohort study	3	2	2	7
Eoh et al. (20)	Korea	Retrospective monocenter cohort study	3	2	3	8
Fang et al. (21)	China	Retrospective monocenter cohort study	3	2	2	7
Fukasawa et al. (22)	Japan	Retrospective monocenter cohort study	3	2	2	7
Nasidius et al. (25)	USA	Retrospective monocenter cohort study	3	2	3	8
Paik et al. (26)	Korea	Retrospective monocenter cohort study	3	1	3	7
Sakai et al. (27)	Japan	Prospective monocenter case-control cstudy	3	1	3	7
Scarabelli et al. (28)	Italy	Prospective non-randomized monocenter cohort study	3	1	3	7
Schwartz et al. (29)	France	Retrospective multicenter cohort study	3	1	2	6
Song and Gao (30)	China	Retrospective monocenter cohort study	2	2	3	7

B. OS Funnel Plot.

B.1 OS Funnel Plot subgroups.

C. DFS Funnel Plot.
